# The Effects of Agomelatine Treatment on Lipopolysaccharide-Induced Septic Lung Injuries in Rats

**DOI:** 10.5152/eurasianjmed.2021.20342

**Published:** 2021-06

**Authors:** Duygu Köse, Tuğba Nurcan Yüksel, Zekai Halıcı, Elif Çadırcı, Muhammed Ali Gürbüz

**Affiliations:** 1 Clinical Research, Development and Design Application, and Research Center, Atatürk University, Erzurum; 2 Department of Pharmacology, Namık Kemal University Faculty of Medicine, Tekirdağ, Turkey; 3 Department of Pharmacology, Atatürk University Faculty of Medicine, Erzurum, Turkey; 4 Department of Histology and Embryology, Atatürk University Faculty of Medicine, Erzurum, Turkey

**Keywords:** Sepsis, agomelatine, tumor necrosis factor-alpha, nuclear factor-kappa B

## Abstract

**Objective:**

We designed an experimental model of sepsis in rats to investigate the effects of agomelatine (AGO) on lung tissues using molecular and histopathological methods.

**Materials and Methods:**

In our experimental model, the 32 rats were divided into 4 groups: group 1: control group (HEALTHY); group 2: lipopolysaccharide group (LPS); group 3: LPS plus 50 mg/kg AGO group (LPS + AGO50); and group 4: LPS plus 100 mg/kg AGO group (LPS + AGO100). An LPS-induced sepsis model was performed to replicate the pathology of sepsis. Rats from all 4 groups were killed after 12 hours, and their lungs were quickly collected. To investigate the therapeutic strategy, we evaluated tumor necrosis factor-alpha (TNF-α) and nuclear factor-kappa B (NF-κB) messenger RNA expressions by real-time polymerase chain reaction using molecular methods and lung tissue damage indicators using histopathological methods.

**Results:**

The expressions of TNF-α and NF-κB were reduced in the groups treated with AGO. The histopathology results supported the molecular results.

**Conclusion:**

In this experimental study, we demonstrated for the first time the positive effects of AGO on LPS-induced sepsis in lung tissue using molecular and histopathological methods, indicating that it contributes to the prevention of lung damage.

## Introduction

Sepsis is a life-threatening condition that can cause death in hospitalized patients.^1^ It is characterized by tissue damage in organs due to the increased permeability of the vascular endothelium, which in turn causes plasma extravasation and bacterial translocation.^2^ In a sepsis infection, the increased levels of bacterial enzymes and toxins caused by a bacterial attack, perfusion disorders, or widespread intravascular coagulation result in damage to the liver, kidneys, heart, intestines, and lungs. Acute organ dysfunction mostly affects the cardiovascular and respiratory systems. Lung injury in an episode is defined as pathologically diffuse alveolar damage and is characterized by noncardiac bilateral infiltrates and hypoxemia.^3^ Various mechanisms, especially inflammatory ones, play a role in giving rise to lung damage observed in sepsis. A septic response is triggered by the overexpression of inflammatory cytokines, such as nuclear factor-kappa B (NF-κB) and tumor necrosis factor- alpha(TNF-α), and increased levels of free radicals.^4^

To create polymicrobial sepsis to better understand the mechanism of clinical sepsis and develop more effective treatments, a wide variety of models are used, including cecal ligation and puncture, mesenteric ischemia, and reperfusion models, and high-dose lipopolysaccharide (LPS) administration.^5^–^7^ Induction of sepsis leads to increased levels of reactive oxygen species (ROS) coupled with an increase in the levels of inflammatory cytokines such as TNF-α and NF-κB increase within 4 hours in the peritoneal cavity, blood, and peripheral organs, which results in tissue damage in many peripheral organs.^8^ Lung damage is considered crucial given its association with poor prognosis in sepsis and should be prevented.

Melatonin is a hormone synthesized from a substance called tryptophan. It is secreted from the pineal gland in the brain in the dark at night. Melatonin has positive effects on asthma bronchiale, chronic obstructive pulmonary disease, infectious respiratory diseases, lung cancer, pleural diseases, and vascular diseases of the lung.^9^ It is known that melatonin has strong anti-inflammatory and antioxidant effects and provides protection against various diseases.^10^,^11^ Three groups of melatonin receptors (MTs) have been defined: MT1, MT2, and MT3.^12^ MT1 and MT2 are expressed in peripheral organs and cells. MT1 and MT2 are membrane receptors and mediate some of the main effects of melatonin.^13^,^14^ MT1 suppresses neuronal firing activity, and MT2 mainly causes phase shifts.^15^ MT1 and MT2 contribute to a variety of immunological effects or vasomotor control. MT2 causes vasodilation, whereas MT1 causes vasoconstriction. The function and distribution of MT3 has not yet been sufficiently elucidated. It has been shown in several studies that the effects of melatonin on the lung are achieved through MT2.

Agomelatine (Valdoxan), henceforth referred to as AGO, is an approved drug for the treatment of major depression in adults and a nonselective agonist for MT1 and MT2 and has anti-inflammatory properties.^16^–^18^ Melatonin is known to alleviate acute lung injury.^19^ The long-term use of melatonin causes hormonal side effects on the chronological rhythm, which is why melatonin cannot be used for a prolonged period. In our study, we investigated the effects of AGO, which does not include the side effects observed with the use of melatonin, on septic lungs.

## Materials and Methods

### Animals

Thirty-two female albino Sprague Dawley rats (10–12 weeks old and 240–260 g) were taken from the Atatürk University Medical Experimental Research Center. The experiments were carried out under normal temperature conditions (22 °C), regular ventilation, and controlled light conditions (12-hour light/dark cycle). The use and care of laboratory animals was approved by the Atatürk University Institutional Animal Care and Use Committee, and the experiments were carried out according to international guidelines (Date, September 17, 2020; Meeting number, 10; Decision number, 143; Document number, 42190979-000-E.2000225779). Rats were housed in plastic cages with a sawdust bottom, and standard rat food and tap water were provided ad libitum.

### Chemicals

LPS (E. coli O55: B5), which is derived from the cell wall of most Gram-negative bacteria, was purchased from Sigma-Aldrich (St. Louis, MO, USA). AGO (Valdoxan 25 mg tab was purchased from Servier, Turkey; ketamine (Ketalar 500 mg/10 mL) from Pfizer, Turkey; and xylazine (Basilazin 2%) from BioTek, Turkey. Metamizole sodium (Novalgin 500 mg/mL injectable preparation) was obtained from Sanofi-Aventis, and all other chemicals for the laboratory experiments were purchased from Sigma and Merck (Germany). Oral drug administration was given by calculating the drug per gram for each rat with the help of a gavage according to the size of the rats. Similarly, in intraperitoneal applications, the amount of drug to be applied per gram was calculated, and drug applications were completed with the help of an injector.

### Experimental Design

In our experimental model, we divided 32 rats into 4 groups to carry out our research;

Group 1 (HEALTHY): Control group
Group 2 (LPS): Rats were administered only LPS
Group 3 (LPS +AGO50): Rats were administered LPS + 50 mg/kg AGO
Group 4 (LPS +AGO100): Rats were administered LPS + 100 mg/kg AGO

AGO at doses of 50 and 100 mg/kg were administered orally 1 hour before intraperitoneal LPS administration.^20^,^21^

### The LPS-Induced Sepsis Model

LPS obtained via phenol extraction from serotypes were used.^22^ All LPS were diluted in sterile pyrogenic physiological serum. To create an experimental polymicrobial sepsis model, LPS was injected intraperitoneally with a dose of 10 mg/kg.^23^ All animals received intraperitoneally 2 mL of normal saline intraoperatively and 6 hours postoperatively for fluid resuscitation. The rats were able to freely access the water but were given no postoperative food for 12 hours until they were killed. Rats from all groups were killed after 12 hours with a general anesthetic (80 mg/kg ketamine + 8 mg/kg xylazine).^24^ The lungs were then quickly removed from all rats. Half of the lung tissue was kept at −80 °C for molecular analysis, and the other half of the lung tissue was fixed in a 10% formalin solution for histopathological analysis.

### Histopathological Analysis

#### Light Microscopy

Lung tissues were collected from all the rats, fixed in 10% neutral formalin for 48 to 72 hours, then routinely processed and embedded in paraffin wax. Serial sections of 3-μm thickness were cut and stained with hematoxylin and eosin (HE) for histopathology before being examined under a light microscope (Olympus BX51, Japan). To evaluate septic lung damage, edema, alveolar wall thickness, and inflammatory cell infiltration were evaluated and scored histopathologically. At least 5 areas were evaluated for each lung slide at ×40 magnification, and the severity of the changes was determined using the following grading: grade 0: − (0% negative), grade 1: + (0%–33% positive), grade 2: ++ (33%–66% moderately positive), and grade 3: +++ (66%–100% severely positive).^4^

#### Molecular Studies

We investigated molecularly the expression of inflammatory mediators such as TNF-α and NF-κB by real-time polymerase chain reaction (RT-PCR). For this, we made the following order process: homogenization of lung tissues, RNA isolation, complementary DNA (cDNA) synthesis, and quantitative determination of mRNA expressions.

### Real-Time PCR

#### RNA Extraction from Rat Lung Tissue

Tissues (20 mg) were homogenized with nitrogen with Tissue Lyser II (Qiagen, Germany). RNA extraction was performed on the QIAcube. The total amount of mRNA was measured at 260 nm using a nanodrop spectrophotometer (EPOCH Take 3 Plate, BioTek USA ). The obtained RNA was stored at −80 °C under the required conditions.

#### Reversed Transcriptase Reaction and cDNA Synthesis

Production of cDNA from RNA was achieved with the High Capacity cDNA Reverse Transcription Kit (Applied Biosystems, USA) according. For cDNA synthesis, 10-μL RNA was used in each reaction with Thermal Cycler (Applied Biosystems, Foster City, CA, USA) according to temperature values. The amount of cDNA was determined by the nanodrop spectrophotometer (EPOCH Take 3 Plate), and the obtained cDNA was stored at −20 ° C. For the cDNA synthesis reaction, 10 μL of total RNA, 2 μL of 10 × Reverse Transcription Buffer, 0.8 μL of a mixture of 25 × Deoxynucleotides, 2 μL of 10 × Reverse TranscriptionRandom Primers, 1 MultiScribe Reverse Transcriptase, and 4.2 μL of diethylpyrocarbonate H_2_O were used.

#### Quantitative Determination of TNF-α and NF-κB mRNA Expressions by RT-PCR

TNF-α and NF-κB mRNA expression was achieved using the TaqMan Gene Expression Master Mix with B-actin (Applied Biosystems, USA) as the reference gene. Amplification and quantification were performed using the StepOnePlus Real-Time PCR System (Applied Biosystems, USA). The following TaqMan Gene Expression Assays for 200 ng cDNA were continued by pipetting, using X μL cDNA (200 ng), 10 μL TaqMan Master Mix, and 1 μL Assay and completed to 20 μL with RNase free H_2_O, for 40 cycles. The cycle threshold (CT) is the cycle number at which the amount of fluorescent signal in RT-PCR experiments exceeds the minimum value (threshold value) required to be observed. CT values were automatically converted into delta Ct (2–ΔΔCt), and the results were statistically evaluated in the SPSS 25.00 program (IBM SPSS Corp.; Armonk, NY, USA).^4^

#### Statistical Analysis

Data are expressed as meansstandard deviation (SD). To test for any difference among the groups, a one-way analysis of variance test and Duncan’s tests were performed. *P* < .05 was accepted as statistically significant.

## Results

### Histopathology Results

#### HE Staining Results

**Figure 1. a-d:**
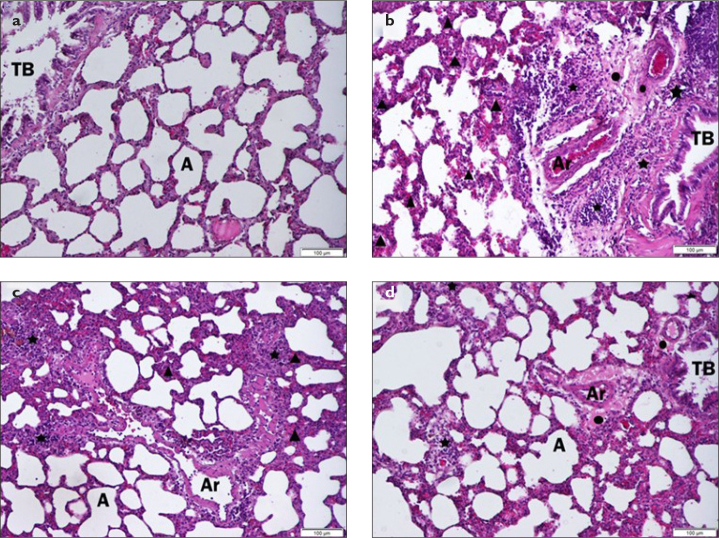
Hematoxylin-eosin staining in the lung tissues of rats in all groups. (a) indicates the HEALTHY group, (b) the LPS group, (c) the LPS + AGO50 group, and (d) the LPS + AGO100 group. LPS: lipopolysaccharide, AGO: agomelatine, TB: terminal bronchiole, A: alveoli, Ar: arteriole, point: edematous areas, star: leukocyte infiltration areas, triangle: alveolar septum thickening. The scale bar for A, C, D, E and F is 100 μm and for B, it is 50 μm.

HEALTHY: The structure of the terminal bronchioles (TB) and alveoli (A), the thickness of the alveolar septa, and the appearance of the arteriolar areas were healthy (Figure 1a).

LPS: General appearance is compatible with sepsis findings. An increase in alveolar wall thickness and vasodilation (triangles) in alveolar capillaries were observed. Leukocyte infiltration was detected in the whole lung parenchyma, although more intense around the pulmonary artery and terminal bronchioles (stars). Edematous areas were observed in the periphery of the pulmonary artery (points). It was observed that alveolar wall integrity was lost, and lung tissue was destroyed in some parts of the section. (Figure 1b)

LPS+ AGO 50/100 mg/kg: Compared with the sepsis group, there were partial non–dose-related signs of improvement in the treatment groups. Edematous areas around the arterioles, leukocyte infiltration spreading to the lung parenchyma, increase in alveolar wall thickness, and vasodilation findings continue (Figure 1c and 1d).

When histopathological findings were examined, it was concluded that the causative agent had a positive effect, albeit limited, in the elimination of sepsis-induced damage in the lung tissue. To better understand the histopathological data, intracomparative scoring was done according to edema and inflammation assets: none (−), mild (+), medium (++), and intense (+++) (tab 1).^4^,^25^

### RT-PCR Results

#### TNF-α and NF-kB mRNA Levels

**Figure 2:**
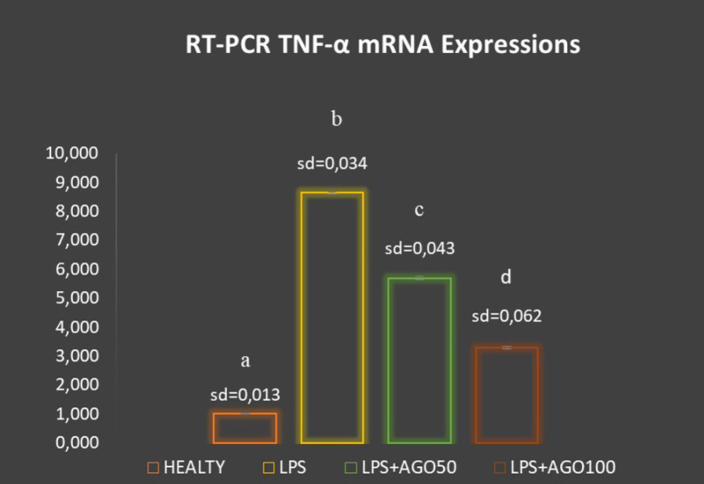
The effects of agomelatine on TNF-α mRNA expressions of lung tissue. Gene expression was detected by quantitative RT-PCR analysis. The results were normalized to b-actin (housekeeping gene control). The relative expression levels were calculated by the 2-ΔΔcT method and were indicated as “fold change” compared with control groups. Means that have the same letter are not significantly different to those of the test of Duncan (*P* = .05). LPS: lipopolysaccharide, AGO: agomelatine, SD: standard deviation, RT-PCR: real-time polymerase chain reaction, TNF-α: tumor necrosis factor α

**Figure 3:**
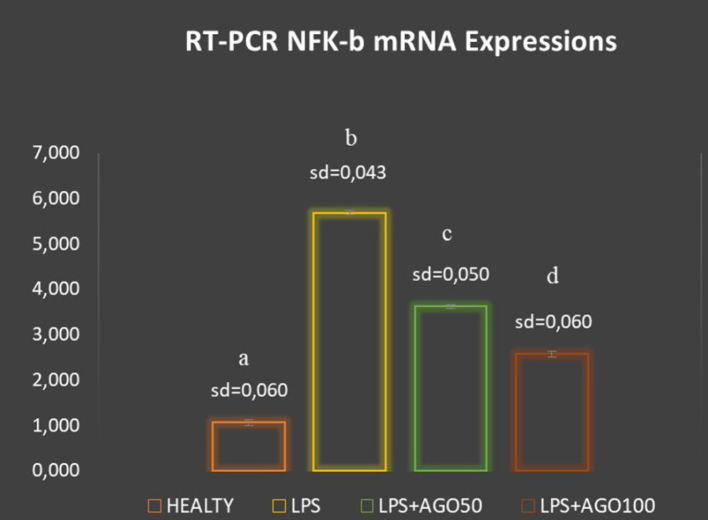
The effects of agomelatine on NF-κB mRNA expressions of lung tissue. Gene expression was detected by quantitative RT-PCR analysis. The results were normalized to b-actin (housekeeping gene control). The relative expression levels were calculated by the 2-ΔΔcT method and were indicated as “fold change” compared with control groups. The results are means ± SD. Means that have the same letter are not significantly different to those of the test of Duncan (P = .05). LPS: lipopolysaccharide, AGO: agomelatine, SD: standard deviation, RT-PCR: real-time polymerase chain reaction, NF-κB: nuclear factor kappa B

Using RT-PCR, we investigated TNF-α and NF-κB mRNA expressions in the rats’ lung tissue. TNF-α and NF-κB mRNA levels were significantly higher in the LPS group than in the HEALTHY group (*P* < .05) (Figure 2 and 3). In contrast, TNF-α and NF-κB mRNA levels were reduced in the AGO treatment groups in a dose-dependent manner. Particularly in the LPS+AGO100 group, it was observed that TNF-α and NF-κB mRNA levels were significantly lower than the LPS group (*P* < .05).

## Discussion

We investigated the effects of AGO on septic lung tissues of rats with an experimental sepsis model. We analyzed lungs using molecular and histopathological methods. We molecularly evaluated TNF-α and NF-kB m RNA expressions by RT-PCR and histopathologically evaluated edema, alveolar wall thickness, and inflammatory cell infiltration of lung tissues.

Sepsis causes an increase in inflammatory mediators such as TNF-α and NF-κB in lung tissue.^8^ In septic conditions, neutrophils and macrophages release TNF-α, giving rise to negative effects on lung tissue .^4^,^8^ İncreased TNF-α causes upregulation of NF-κB. NF-κB also causes upregulation of many inflammatory genes, including TNF-α.^8^ This vicious circle causes increases ROS, chemokine, and cytokine formation.^4^ We also found that the expressions of both TNF-α and NF-kB increased in the sepsis group. We found that TNF-α and NF-kB expressions were lower in the AGO treatment groups than in the sepsis group. We showed that AGO inhibits the increased levels of TNF-α and NF-κB by LPS in the lung depending on the dose. This inhibition may be caused by anti-inflammatory effects due to melatonin agonism.^10^ Melatonin’s anti-inflammatory effects are known to be through the NF-κB signaling pathway, and its molecular activity is due to the inhibition of NF-κB.^26^ It is also usual to have TNF-α inhibition following NF-κB inhibition.

It was observed that increased alveolar wall thickness and cellular infiltration in the lungs in the LPS group decreased in the Ago treatment groups.We found in histopathological examination that the occurrence of lung damage was less in the groups that received AGO treatment. The positive effects of AGO, a peripheral melatonin agonist, on the lung may be due to melatonin’s anti-inflammatory, vascular regulatory, and antioxidant properties.^10^,^17^,^27^,^28^

In this study, we demonstrated the molecular and histopathological effects of AGO on LPS-induced sepsis for the first time and revealed its protective efficacy. It remains necessary to conduct detailed research on the effects of AGO on sepsis.

In conclusion, this is the first report to demonstrate that AGO prevents lung damage in sepsis. Thus, the use of AGO may be preferred as antidepressant therapy to protect lung tissue in patients at risk of developing sepsis. However, further research is required.

**Main Points**

- In an experimental sepsis model, we investigated molecularly and histopathologically, the effects of agomelatine (AGO), a peripheral melatonin agonist that was found to be suitable for long-term use without central side effects on the lung tissues of rats.
- To investigate the therapeutic strategy, we evaluated tumor necrosis factor α (TNF-α) mRNA expressions and nuclear factor kappa B (NF-κB), by real-time polymerase chain reaction molecularly, and edema, alveolar wall thickness, and inflammatory cell infiltration histopathologically.
- The expressions of TNF-α and NF-κB were reduced in the groups treated with AGO.
- The histopathology results supported the molecular results. We showed for the first time the effects of AGO on experimentally induced sepsis—molecularly and histopathologically—and showed that it contributes to the prevention of lung damage.
- AGO may be preferred as antidepressant therapy to protect lung tissue in patients with the possibility of developing sepsis. However, further research is required.

## Figures and Tables

**Table 1:** Histopathological Scoring Results

Groups	Edema Area	Alveolar Wall Thickness	Inflammatory Cell Infiltration
Healty	−	−	−
LPS	+++	+++	+++
LPS + AGO 50	++	++	++
LPS + AGO 100	+++	++	+

Grade 0: − (0% negative), grade 1: + (0%–33% mild positive), grade 2: ++ (33%–66% moderately positive), grade 3: +++ (66%–100% severely positive).

LPS: lipopolysaccharide, AGO: agomelatine
